# Jintiange capsule may have a positive effect on pain relief and functional activity in OVCF patients with PVA: A meta-analysis of randomized control trials

**DOI:** 10.1097/MD.0000000000038192

**Published:** 2024-05-31

**Authors:** Ningning Feng, Jianbin Guan, Xing Yu, Wenhao Li, Ziye Qiu, Guozheng Jiang, Yongdong Yang

**Affiliations:** a Department of Orthopedics, Dongzhimen Hospital Affiliated to Beijing University of Chinese Medicine, Beijing, China; b Department of Orthopedics, Honghui-Hospital, Xi’an Jiaotong University, Xi’an, China.

**Keywords:** bone mineral density, Jintiange capsule, OVCF, percutaneous vertebral augmentation, systematic review

## Abstract

**Background:**

This study aims to systematically evaluate the clinical efficacy and adverse reactions associated with Jintiange capsule (JTG capsule)-assisted percutaneous vertebral augmentation (PVA) in the treatment of osteoporotic vertebral compression fracture (OVCF).

**Methods:**

A comprehensive search was conducted across multiple databases including PubMed, Cochrane Library, EMBASE, Web of Science Database, China Biomedical Database, China VIP Network, China National Knowledge Infrastructure, Wanfang, and VIP Chinese Journal databases until June 1, 2022. Manual searches were also performed in relevant journals. Randomized controlled trials investigating the efficacy of JTG capsule-assisted PVA in the treatment of OVCF were identified and selected for inclusion. The quality of the included studies was assessed using the Cochrane risk bias assessment tool and Jadad scale. Meta-analysis was conducted using Stata MP18 software.

**Results:**

A total of 138 literatures were retrieved, and 12 RCTS were finally included after screening, involving 1099 patients. Overall, the quality of the included literature was low, and all the included literatures were randomized controlled experiments, among which 9 were grouped by random number table, and 3 did not specify the random assignment plan. The total effective rate of the experimental group was higher than that of the control group (relative ratio: 1.19, 95% confidence interval: 1.11, 1.26, *P* = .868, *I*^2^ = 0%). The heterogeneity of visual analog score, Oswestry disability index, bone mineral density (BMD) of lumbar vertebrae, BMD of femoral neck and bone-γ-carboxyglutamic acid-containing protein was high. The reasons for the high heterogeneity were the age of patients, the follow-up time and the small sample size. There is publication bias in visual analog score, Oswestry disability index scores, and lumbar spine bone mineral density, and we believe that publication bias may be related to selective reporting of positive results by the authors and selective publication of positive results by the publishers.

**Conclusion:**

JTG capsule has demonstrated promising outcomes in alleviating the pain experienced by OVCF patients following PVP. Additionally, it has shown efficacy in enhancing postoperative lumbar and back function. Furthermore, JTG capsule has been associated with improvements in postoperative vertebral BMD and serum bone-γ-carboxyglutamic acid-containing protein levels. These findings suggest that JTG capsule could potentially serve as a viable adjunctive treatment option for managing osteoporosis following PVA.

## 1. Introduction

Osteoporotic vertebral compression fracture (OVCF) is characterized by the onset of pain in various regions of the vertebral body, encompassing the cervical, thoracic, and lumbar vertebrae. Patients with osteoporosis may experience symptoms such as kyphosis, hunchback, and restricted mobility, often triggered by minor trauma or even without apparent trauma. This condition manifests as vertebral body compression resulting from decreased bone density and strength associated with osteoporosis, leading to structural compromise and functional impairment of the spine.^[1]^ OVCF represents a focal bone tissue anomaly stemming from systemic osteoporosis, serving as a poignant indicator of diminished bone strength and a consequential outcome of osteoporosis. As modern society undergoes an aging demographic shift, the incidence of OVCF continues to rise steadily. Delayed diagnosis and treatment of OVCF can precipitate complications such as fracture nonunion, pronounced vertebral wedge deformities, and kyphosis. Timely recognition and intervention are crucial to mitigate these adverse sequelae and optimize patient outcomes. According to the existing literature, the permanent disability rate associated with OVCF can be as high as 50%, significantly impacting the quality of life of affected individuals. Additionally, the mortality rate attributed to complications related to OVCF has been reported to reach up to 20%.^[2]^ These alarming statistics underscore the substantial burden imposed by OVCF on patients, highlighting the urgent need for effective management strategies to mitigate disability and mortality risks and enhance overall quality of life. For patients suffering from OVCF, percutaneous vertebral augmentation (PVA) stands out as a swift and effective intervention. PVA offers rapid pain relief, facilitates the restoration of vertebral height, and minimizes surgical trauma. This approach enables patients to resume mobility promptly, thereby reducing the need for prolonged bed rest and mitigating associated complications. Consequently, PVA has emerged as a common and advantageous treatment modality for managing fresh OVCF, particularly when combined with systemic anti-osteoporosis therapy to address the underlying bone fragility.^[3,4]^ Following PVA in patients with OVCF, the injection of bone cement indeed alters the internal structure and mechanical properties of the fractured vertebral body. This can result in stress concentration within the treated vertebral body as well as the adjacent vertebral bodies. Consequently, there is a risk of refracture in the operated vertebral body and even adjacent vertebral fractures due to the altered biomechanics.^[5]^ Furthermore, it is essential to recognize that surgical interventions alone do not address the underlying BMD issues in patients with OVCF. Consequently, ongoing and regular anti-osteoporosis treatment remains particularly crucial for patients post-vertebral augmentation.^[6]^

In recent years, Chinese medicine has made significant strides in the treatment of osteoporosis.^[7]^ Among these advancements, Jintiange capsule (JTG capsule) emerges as a noteworthy achievement in our country’s independent research and development of new drugs. It stands as the first artificial tiger bone preparation product, sharing similar ingredients and pharmacological effects with natural bones. Modern studies have demonstrated its robust efficacy in strengthening bones, alleviating kidney pain, and promoting bone micro-structure reconstruction and repair.^[8]^ Research indicates that the efficacy of JTG capsule in combating osteoporosis stems from its composition, particularly its high calcium content and rich array of vital components such as collagen and various bone growth factors. Moreover, it contains organic calcium, phosphorus, strontium, magnesium, zinc, and other trace elements crucial for bone formation. These elements collectively promote bone density, inhibit bone resorption, and foster bone formation.^[9,10]^ Despite the widespread clinical utilization of JTG capsule, its effectiveness and potential adverse reactions following vertebral augmentation for OVCF have not been systematically analyzed. Thus, we undertook a meta-analysis to comprehensively evaluate the clinical efficacy and safety profile of JTG capsule-assisted vertebral augmentation for OVCF.

## 2. Materials and methods

This study adhered to the guidelines outlined in the Preferred Reporting Items for Systematic Reviews and Meta-Analyses (PRISMA) statement^[11]^ and followed the methodology outlined in the Cochrane Handbook.^[12]^ Since the study involved a systematic evaluation of existing published literature, ethical approval was not deemed necessary.

### 2.1. Selection of studies

Inclusion criteria for analysis comprised studies meeting the following conditions: study design: randomized controlled trials; study population: patients diagnosed with OVCFs; intervention objectives: comparing clinical outcomes between JTG capsules combined with other pharmaceuticals or therapies subsequent to percutaneous vertebroplasty; outcome measures: studies reporting at least one of the following outcomes: total effective rate, visual analog scale (VAS) score, Oswestry disability index (ODI), bone mineral density (BMD), adverse events, and bone Gla protein (BGP). Studies failing to meet the aforementioned criteria were excluded from the selection process.

### 2.2. Types of interventions

The experimental group underwent treatment with either JTG capsules alone or in conjunction with conventional medication. The prescribed dosage consisted of 3 capsules administered 3 times daily, with a treatment duration spanning 4–12 weeks. Meanwhile, the control group received conventional medicine therapy.

### 2.3. Data extraction and quality assessment

Two reviewers independently assessed study eligibility, with a third investigator involved to arbitrate any discrepancies. Relevant data extracted from the studies included: title; author(s); year of publication; sample size; gender distribution; intervention type; surgical technique utilized; and duration of follow-up.

The study’s quality was assessed independently by 2 researchers following the guidelines outlined in the Cochrane Handbook for Systematic Reviews of Interventions.^[13]^ Evaluation criteria included: random sequence generation, allocation concealment, blinding of participants and personnel, blinding of outcome assessments, incomplete outcome data, selective reporting, and other potential biases. Each study was categorized as low risk, high risk, or unclear for each criterion. In cases of disagreement, the opinion of a third researcher was sought to reach a consensus.

### 2.4. Search strategy

Two researchers systematically conducted electronic searches across multiple databases, including PubMed, Cochrane Library, EMBASE, Web of Science database, Chinese Biomedical Database (CBM), Chinese VIP Information, China National Knowledge Infrastructure (CNKI), and WanFang. The searches encompassed the inception of each database up to June 1, 2022. In instances of disagreement between the 2 researchers, a third researcher provided resolution. The search strategy employed in PubMed, which was adjusted for other databases, involved the following terms: (Jintiange capsule or Jintian ge capsule or Jintiange Jiaonang or Jin Tian Ge Jiaonang or artificial tiger powder) and (bone density or musculoskeletal diseases or OVCF or osteoporosis).

Furthermore, the researchers manually searched the reference lists of all identified articles to identify additional relevant studies. Integration and removal of duplicate trials were conducted using EndNote software.

### 2.5. Data analysis

All the meta-analyses were performed with the Stata MP18. For the pooled effects, mean difference (MD) or standard mean difference (SMD) was calculated for continuous variables according to the consistency of measurement units, and relative ratio (RR) was calculated for dichotomous variables. Continuous outcomes are presented as mean differences and 95% confidence intervals (CI), whereas dichotomous outcomes are presented as relative risk and 95% CI. Heterogeneity was tested using Chi-square test and quantified by calculating *I*^2^ statistic, for which *P* < .05 and *I*^2^ > 50% were defined as high heterogeneity and assessed by the random-effects model. When the Chi-squared test *P* value was >.05 and *I*^2^ tests value was ≤50%, it was defined as an acceptable heterogeneity data and assessed by the fixed-effects model.

## 3. Results

### 3.1. Search results

**Figure 1.**
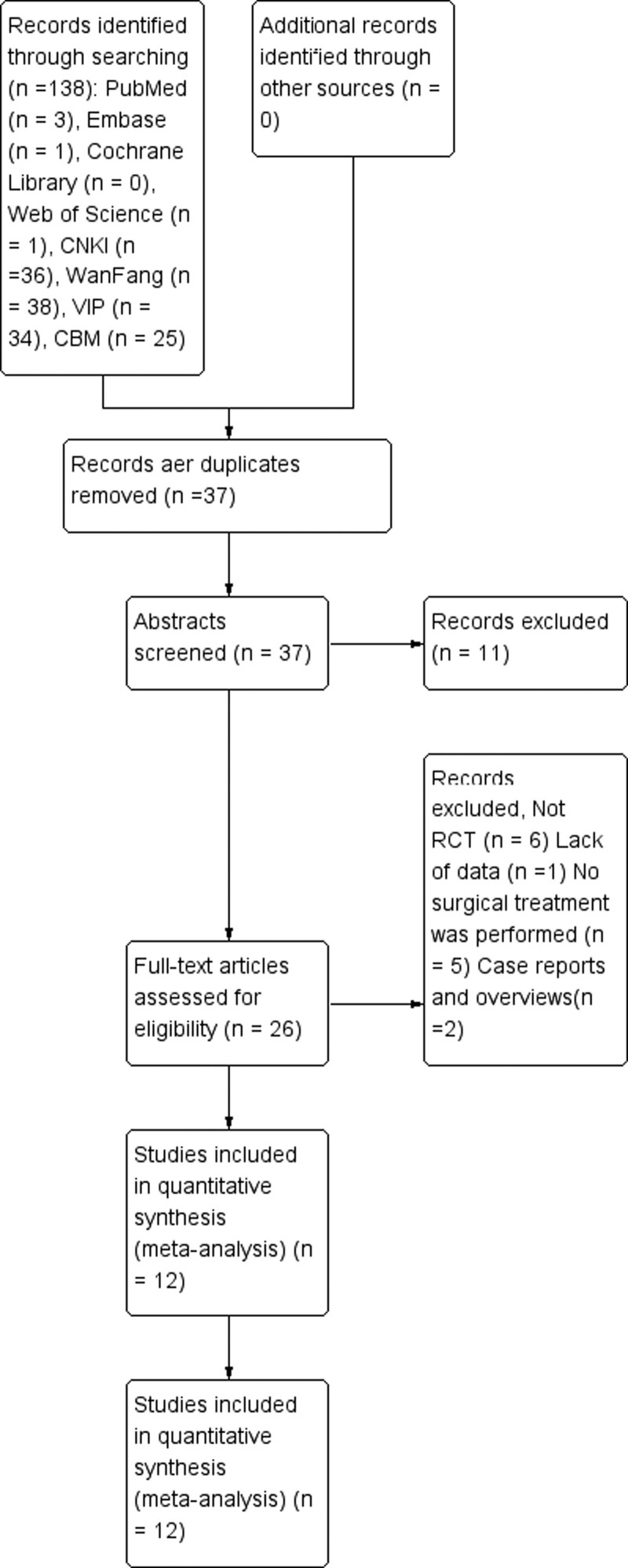
The inclusion process of the literature.

Acting by the search strategy, 138 references were identified. After excluding duplicate studies, 37 studies were scanned based on their abstracts and titles. Then, 26 articles were evaluated by full text. After the full manuscript was assessed, ten records were excluded with the following reasons: not RCT (n = 6), lack of outcomes (n = 1) and no surgical treatment was performed (n = 5). Eventually, 12 studies were included in this meta-analysis (Table 1). The PRISMA statement flow chart shows this process (Fig. 1).

**Table 1:** The basic characteristics of the included studies.

Trail	Simple/size (T/C)	Gender (male/female)	Age (yr), Mean ± SD or median (range)	T	C	Duration (mo)	Main outcomes
Duan^[14]^	144 (72/72)	102/44	T (66.58 ± 2.15) C (67.37 ± 3.04)	JTG capsules	N	3	①②⑤
Yu et al^[15]^	69 (35/34)	37/32	T (65.88 ± 6.35) C (64.72 ± 5.51)	JTG capsules	N	3	②③④⑥
Tan et al^[16]^	104 (52/52)	36/68	67 (57–75)	JTG capsules	Calcium and vitamin D3	6	④
Li et al^[17]^	136 (68/68)	60/76	T (76.6 ± 8.4) C (75.9 ± 8.9)	JTG capsules	Zoledronic acid calcium and vitamin D3	12	①②④
Li^[18]^	60 (30/30)	33/27	T (72.5 ± 5.2) C (73.0 ± 5.3)	JTG capsules	Alendronate sodium + calcium and vitamin D3	6	①②④⑥
Huang^[19]^	58 (29/29)	29/29	T (70.21 ± 6.58) C (70.85 ± 6.49)	JTG capsules	Alendronate sodium + calcium and vitamin D3	12	①②③④⑥
Huang and Pang^[20]^	128 (64/64)	53/75	T (71.8 ± 2.91) C (70.0 ± 2.74)	JTG capsules	calcium and vitamin D3	6	④⑦
Ye^[21]^	90 (45/45)	34/56	T (71.4 ± 9.1) C (70.6 ± 8.7)	JTG capsules	N	3	①④⑦
Wu et al^[22]^	78 (39/39)	34/44	T (66.15 ± 8.12) C (65.37 ± 8.28)	JTG capsules + C	Salmon calcitonin nasal spray + alendronate sodium + calcium and vitamin D3	6	①②④⑤⑦
Han et al^[23]^	80 (40/40)	16/64	T (68.8 ± 5.7) C (70.2 ± 6.2)	JTG capsules	Calcium and vitamin D3	6	②③④⑤
He et al^[24]^	86 (43/43)	43/43	T (66.42 ± 8.82) C (65.22 ± 6.78)	JTG capsules	Calcium and vitamin D3	1	①⑥
Yang^[25]^	66 (33/33)	35/31	T (70.32 ± 1.52) C (70.31 ± 1.55)	JTG capsules + C	Alendronate sodium + calcium and vitamin D3	6	④⑥⑦

C = control group, JTG = Jintiange capsule, N = no treatment, T = trial group. ① = The total effective rate, ② = the VAS score, ③ = ODI, ④ = the BMD of lumbar vertebrae, ⑤ = the BMD of femoral, ⑥ = adverse, ⑦ = BGP.

A total of 1099 participants were randomized into experimental groups (n = 550) and control groups (n = 549). The ethnicity of all participants was Chinese. Moreover, all the studies enrolled OVCF patients.

### 3.2. Risk of bias assessment

**Figure 2.**
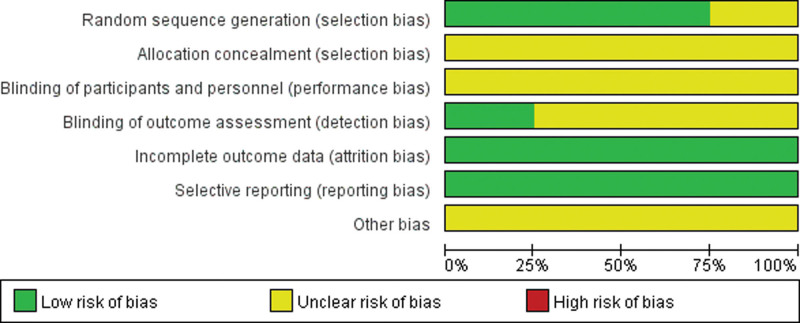
Risk of bias assessment in studies.

**Figure 3.**
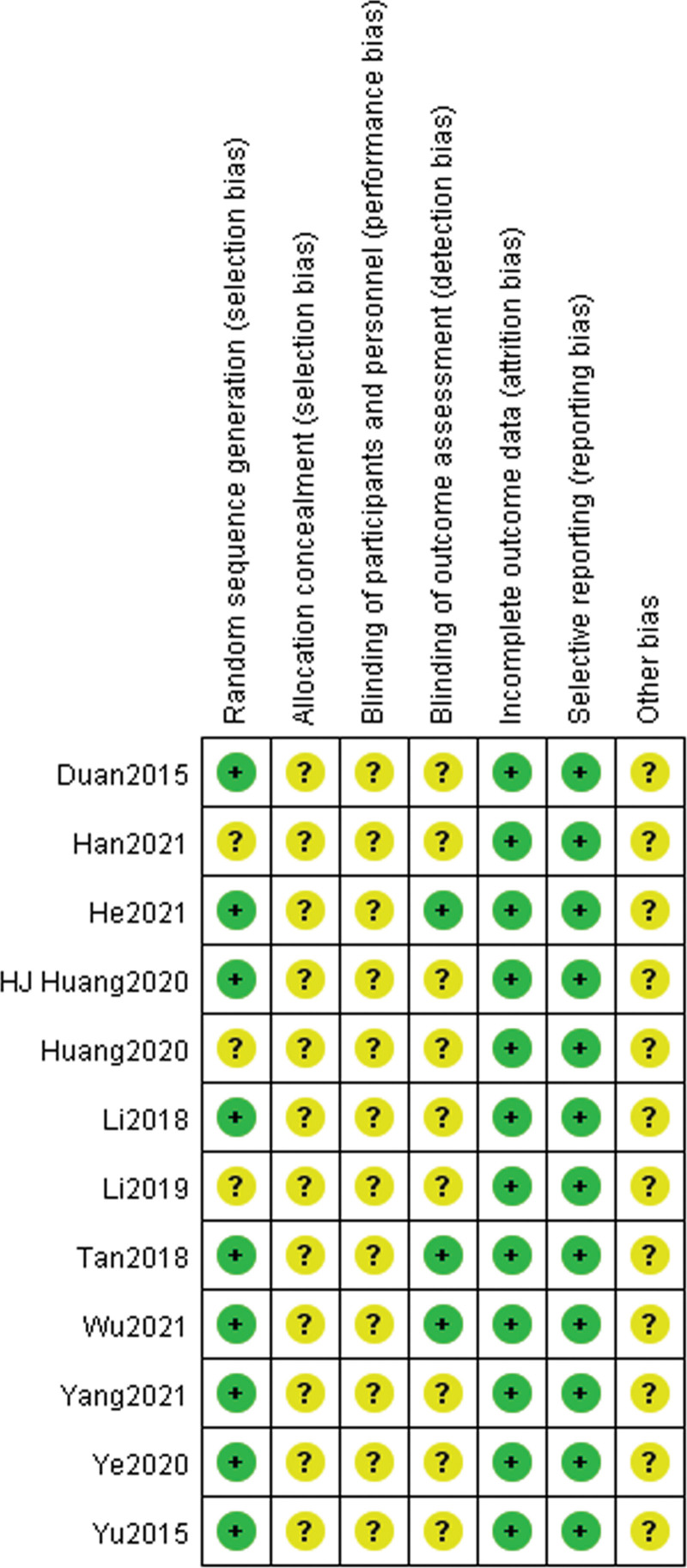
Risk of bias assessment for each included study in the review.

In general, the methodological quality of the included trials may not be high enough (Figs. 2 and 3). All of the 12 included studies involved 2-arm designs and were declared as random controlled trials, and 9 trials reported proper generation methods (random number table or coin toss) with a low risk of bias.^[14–17, 19, 21, 22, 24, 25]^ Three trials did not describe the randomization procedure clearly.^[18, 20, 23]^ Three trials reported the blinding of outcome assessment.^[16, 22, 24]^ None of trials reported the concealed allocation method of patients and investigators. None of the trials reported any blinding of patients and investigators.

### 3.3. Primary outcomes

#### 3.3.1. Total effective rate

**Figure 4.**
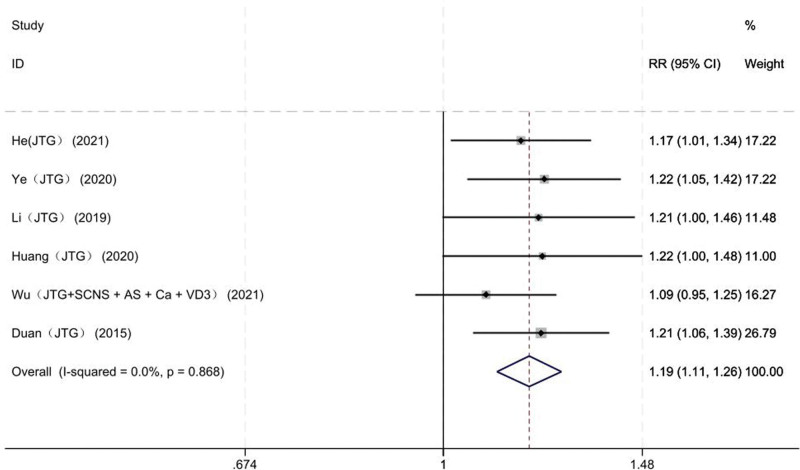
Forest plot showing the total effective rate between JTG capsule group and the Western medicine group. AS = alendronate sodium, Ca = calcium, JTG = Jintiange capsule, SCNS = salmon calcitonin nasal spray, VD3 = vitamin D3.

Six studies on 258 patients reported the total effective rate of the JTG capsule group and the Western medicine group. Meta-analysis showed that the total effective rate of the JTG capsule group was significantly higher (RR: 1.19; 95% Cl: 1.11, 1.26; *P* = .868, *I*^2^ = 0%) than that of the Western medicine group. We chose the fixed-effects model to analyze the incidence of the total effective rate (Fig. 4).

#### 3.3.2. Adverse effect

**Figure 5.**
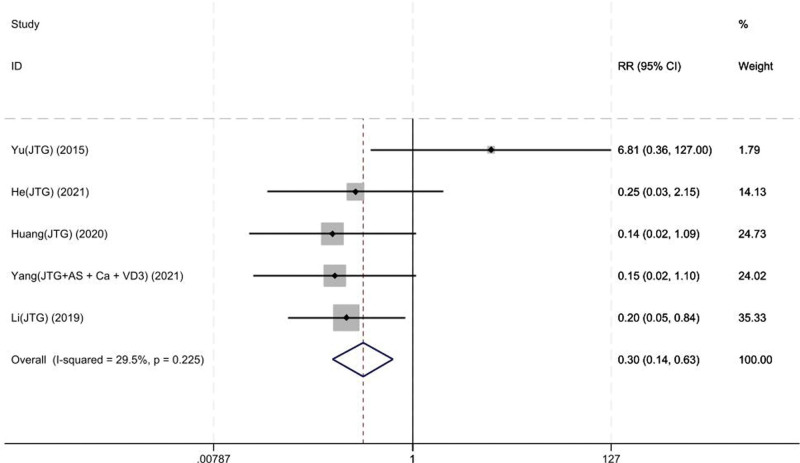
Forest plot showing the risk ration of the adverse effect between the JTG capsule group and the Western medicine group. AS = alendronate sodium, Ca = calcium, JTG = Jintiange capsule, VD3 = vitamin D3.

Five studies on 170 patients reported the adverse effects of the JTG capsule group and the Western medicine group. Based on the chi-squared test *P* value (*P* = .225 and *I*^2^ test value (*I*^2^* *= 29.5%), we chose the fixed-effects model to analyze the incidence of cement leakage. The pooled results showed that compared with the Western medicine, JTG capsule group significantly decreased the risk of adverse effects (RR: 0.30; 95% CI: 0.14–0.63, Fig. 5).

#### 3.3.3. VAS scores

**Figure 6.**
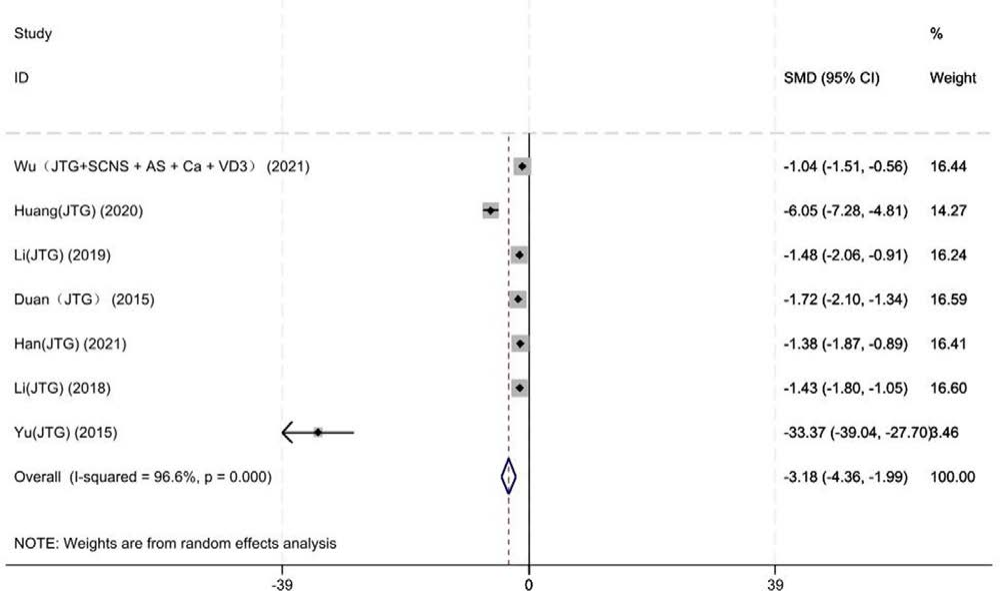
Forest plot showing the VAS scores between the JTG capsule group and the Western medicine group. AS = alendronate sodium, Ca = calcium, JTG = Jintiange capsule, SCNS = salmon calcitonin nasal spray, VAS = visual analog score, VD3 = vitamin D3.

Seven studies on 313 patients provided preoperative and final follow-up VAS scores. Based on the chi-squared test *P* value (*P* = .000 and *I*^2^ test value (*I*^2^ = 96.6%), we chose the random-effects model to analyze the SMD of VAS scores between the JTG capsule group and the Western medicine group. The pooled results showed that compared with the Western medicine, JTG capsule group significantly decreased the VAS scores (SMD: −3.18; 95% CI: −4.36 to −1.99, Fig. 6).

#### 3.3.4. ODI scores

**Figure 7.**
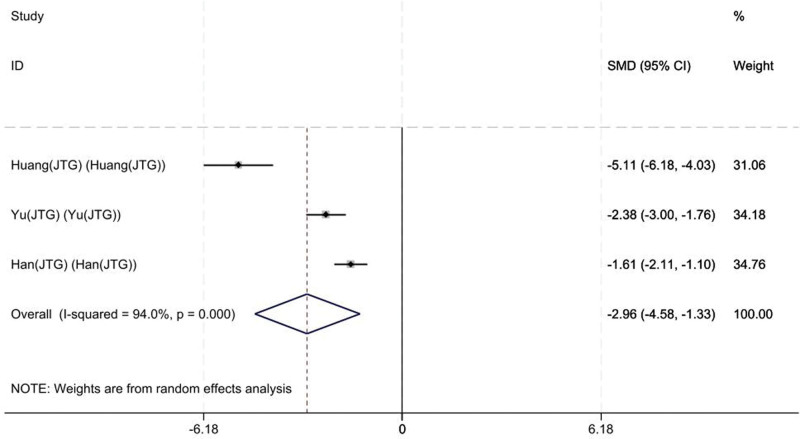
Forest plot showing the ODI scores between the JTG capsule group and the Western medicine group. JTG = Jintiange capsule, ODI = Oswestry disability index.

Three studies on 104 patients provided preoperative and final follow-up ODI scores. Based on the chi-squared test *P* value (*P* = .000 and *I*^2^ test value (*I*^2^ = 94%)), we chose the random-effects model to analyze the SMD of ODI scores between the JTG capsule group and the Western medicine group. The pooled results showed that compared with the Western medicine, JTG capsule group significantly decreased the ODI scores (95% CI: −4.74 to −1.32, Fig. 7).

#### 3.3.5. BMD of the lumbar vertebrae

**Figure 8.**
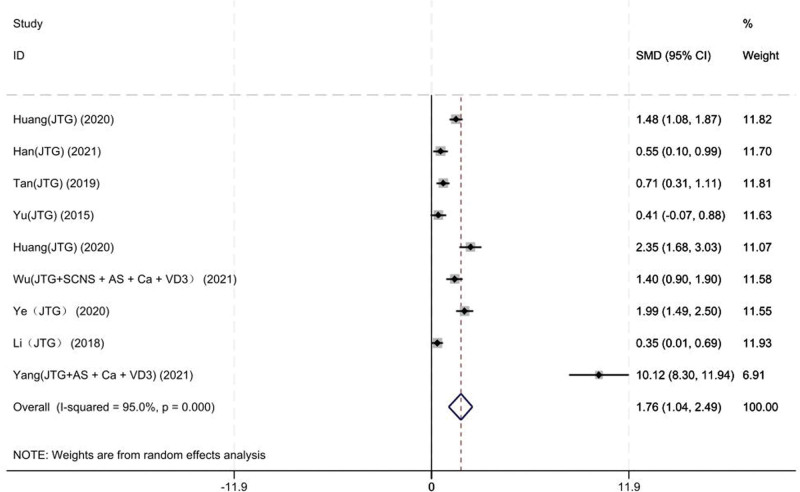
Forest plot showing the BMD of the lumbar vertebrae between the JTG capsule group and the Western medicine group. AS = alendronate sodium, BMD = bone mineral density, Ca = calcium, JTG = Jintiange capsule, SCNS = salmon calcitonin nasal spray, VD3 = vitamin D3.

Nine studies on 405 patients provided preoperative and final follow-up BMD of the lumbar vertebrae. Based on the chi-squared test *P* value (*P* = .000 and *I*^2^ test value (*I*^2^ = 95%), we chose the random-effects model to analyze the MD of BMD of the lumbar vertebrae between the JTG capsule group and the Western medicine group. The pooled results showed that compared with the Western medicine, JTG capsule group significantly increased the BMD of the lumbar vertebrae (95% CI: 1.04–2.49, Fig. 8).

#### 3.3.6. BMD of the femoral neck

**Figure 9.**
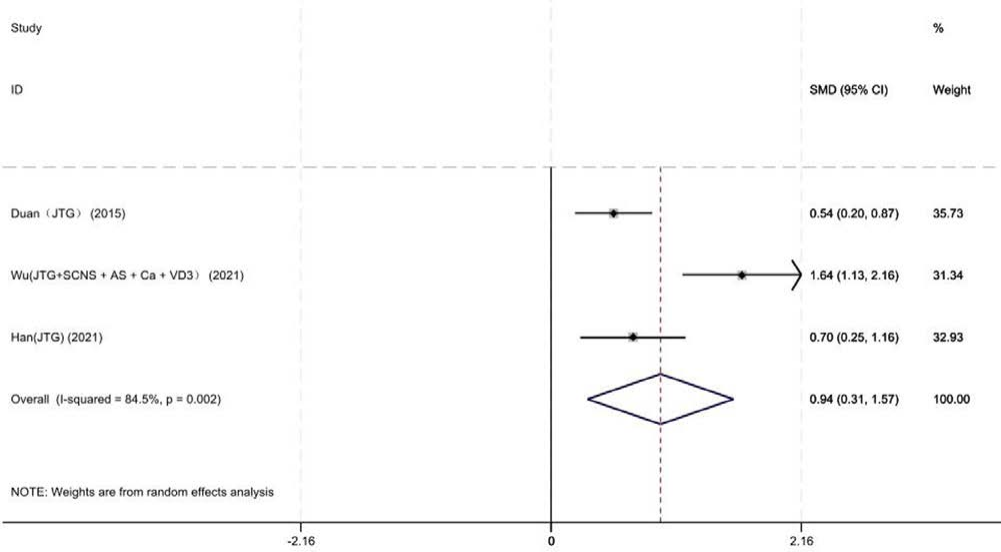
Forest plot showing the BMD of the femoral neck between the JTG capsule group and the Western medicine group. AS = alendronate sodium, BMD = bone mineral density, Ca = calcium, JTG = Jintiange capsule, SCNS = salmon calcitonin nasal spray, VD3 = vitamin D3.

Three studies on 151 patients provided preoperative and final follow-up BMD of the lumbar vertebrae. Based on the chi-squared test *P* value (*P* = .002 and *I*^2^ test value (*I*^2^ = 84.5%)), we chose the random-effects model to analyze the MD of BMD of the femoral neck between the JTG capsule group and the Western medicine group. The pooled results showed that compared with the Western medicine, JTG capsule group significantly increased the BMD of the femoral neck (95% CI: 0.04–0.14, Fig. 9). After Wu 021 was removed, the heterogeneity became 0%.

#### 3.3.7. BGP level

**Figure 10.**
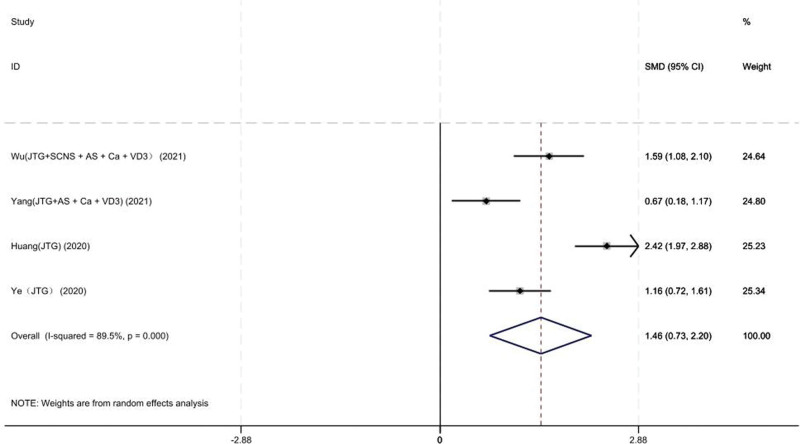
Forest plot showing the BGP level between the JTG capsule group and the Western medicine group. AS = alendronate sodium, BGP = bone-γ-carboxyglutamic acid-containing protein, Ca = calcium, JTG = Jintiange capsule, SCNS = salmon calcitonin nasal spray, VD3 = vitamin D3.

Four studies on 181 patients provided preoperative and final follow-up BGP levels. Based on the chi-squared test *P* value (*P* = .000 and *I*^2^ test value (*I*^2^ = 89.5%)), we chose the random-effects model to analyze the SMD of BGP level between the JTG capsule group and the Western medicine group. The pooled results showed that compared with the Western medicine, JTG capsule group significantly increased the BGP level (95% CI: 0.73–2.20, Fig. 10).

### 3.4. Publication bias

**Figure 11.**
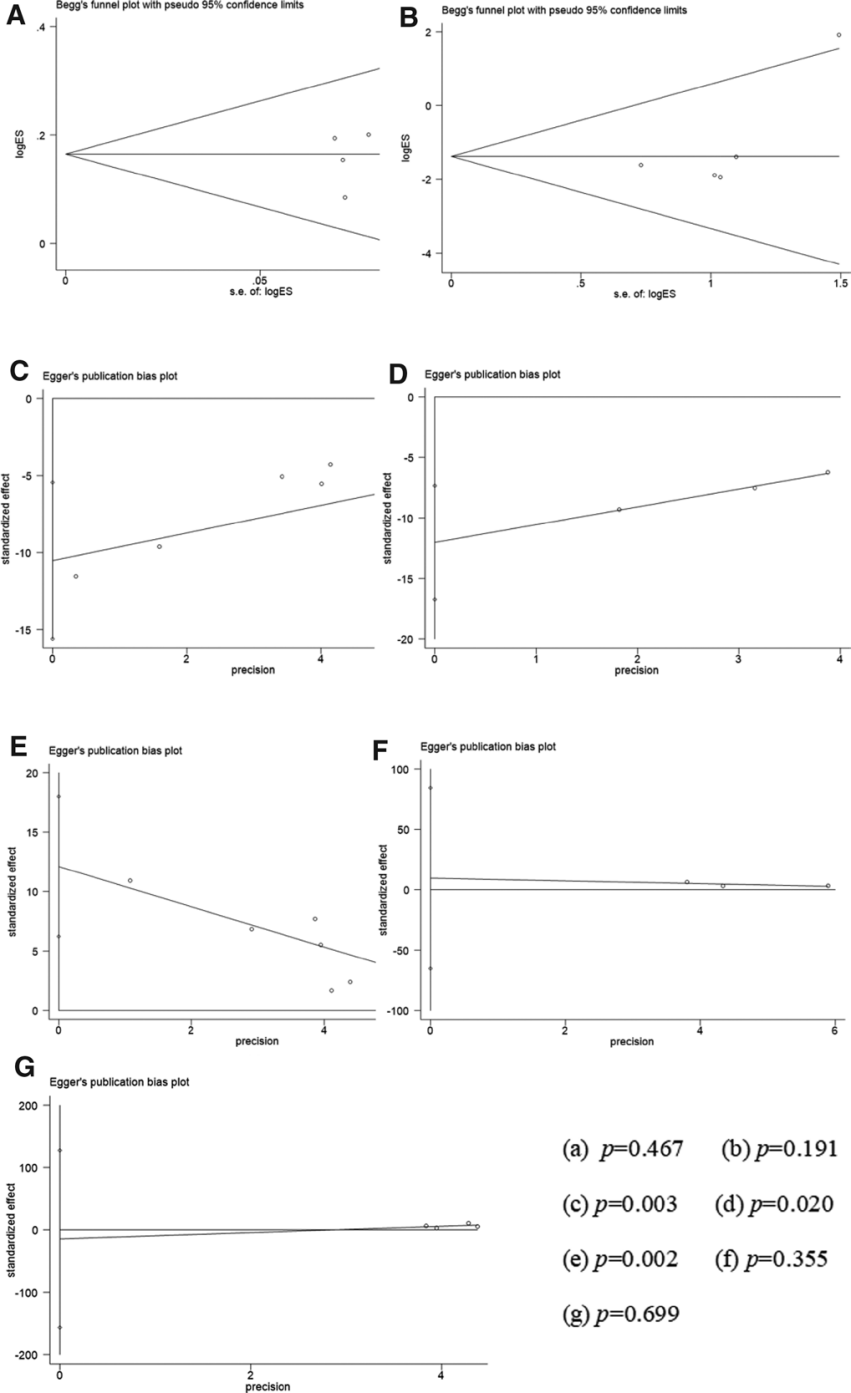
Funnel plot of studies included in the meta-analysis.

The BMD of the lumbar vertebrae is the common outcome index of 9 studies, and it is also the main indicator for evaluating the 2 groups. Therefore, this outcome index was used to make a funnel plot to detect publication bias, as shown in Figure 11. Visual inspection of the funnel plots showed symmetry, suggesting that there was no publication bias.

Among our outcome indicators, the total effective rate and adverse effects were dichotomous variables, so the Begg method was used to detect publication bias, and the VAS, ODI score, lumbar spine BMD, femoral neck BMD, and BGP were continuous variables, so the Egger method was used to detect publication bias. The test results showed that the total effective rate (*P* = .467), adverse reactions (*P* = .191), VAS (*P* = .003), ODI (*P* = .020), lumbar spine bone mineral density (*P* = .002), femoral neck bone mineral density (*P* = .355), and BGP (*P* = .699), with publication bias for the VAS, ODI scores and lumbar spine bone mineral density (*P* < .05). JTG capsule is similar in composition to natural tiger bone, which has the effect of strengthening the tendons and bones, dispersing cold and relieving pain, and has a wide range of clinical applications (Fig. 11).

### 3.5. Sensitivity analysis

**Figure 12.**
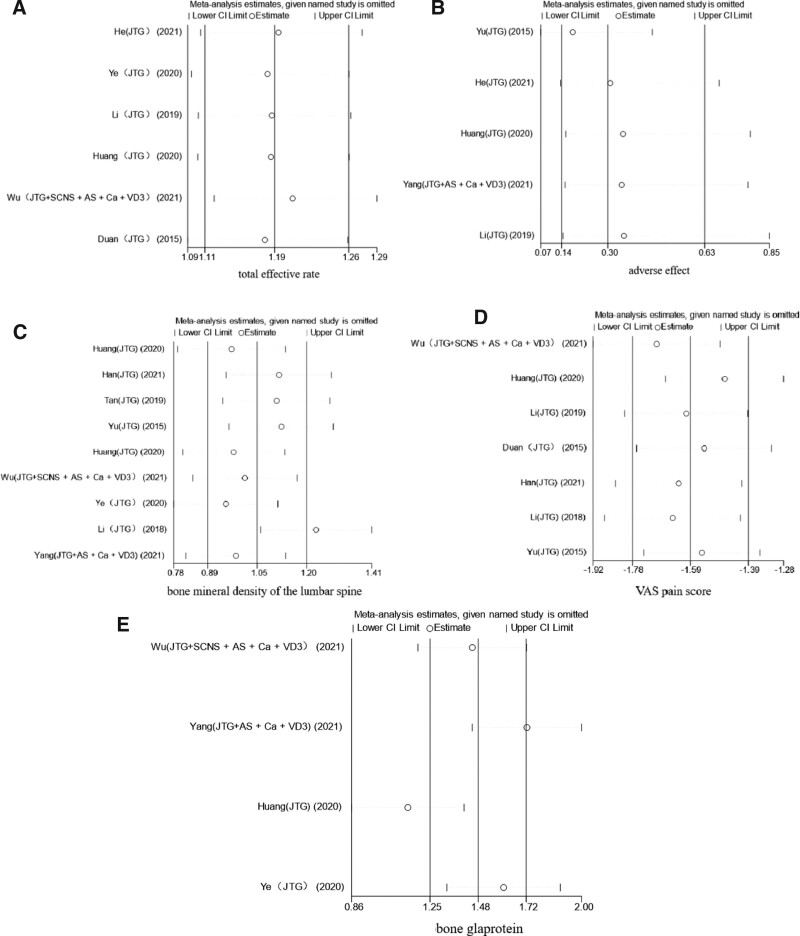
Sensitivity analysis results graph. AS = alendronate sodium, Ca = calcium, JTG = Jintiange capsule, SCNS = salmon calcitonin nasal spray, VD3 = vitamin D3.

We performed sensitivity analysis on the main evaluation indexes, total effective rate, adverse effects, lumbar spine bone density, VAS score, and BGP by excluding the literature one by one, and drew a sensitivity analysis diagram, from which it can be seen that after excluding the literature one by one and then combining the effect sizes, the results of the combined results did not undergo any significant changes, which indicates that the combined results are robust and reliable (Fig. 12).

### 3.6. Heterogeneity analysis

**Figure 13.**
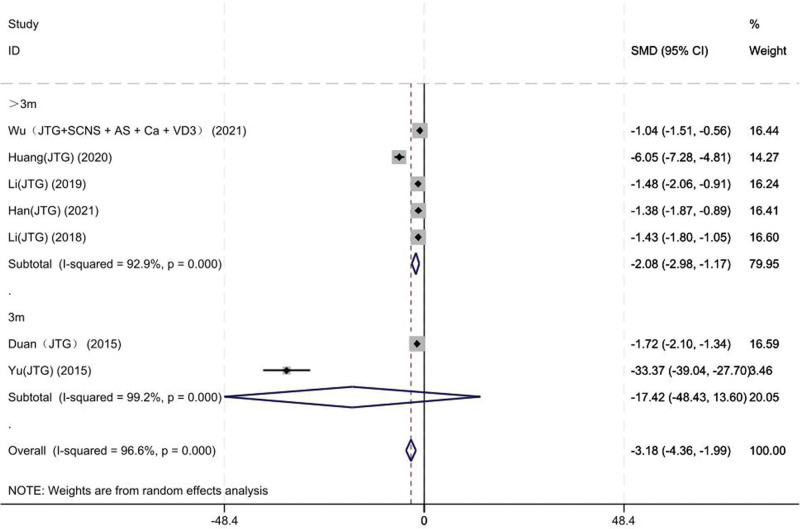
Forest plot for subgroup analysis of follow-up time. AS = alendronate sodium, Ca = calcium, JTG = Jintiange capsule, SCNS = salmon calcitonin nasal spray, VD3 = vitamin D3.

The heterogeneity of VAS scores (*I*^2^ = 96.6%), ODI scores (*I*^2^ = 94%), BMD of the lumbar vertebrae (*I*^2^ = 95%), BMD of the femoral neck (*I*^2^ = 84.5%) and BGP (*I*^2^ = 89.5%) level is high. Subgroup analysis of VAS scores was performed for the mean baseline of the follow-up 3 months and more than 3 months. In 2 studies,^[14, 15]^ follow-up time baseline levels were 3 months, which in the remaining 5 studies^[17–19, 22, 23]^ were, respectively, 12, 6, 12, 6, and 6 months. The heterogeneity analysis suggested that there was lower heterogeneity after subgroup analysis. The results suggested that the follow-up time may be a source of heterogeneity (Fig. 13).

**Figure 14.**
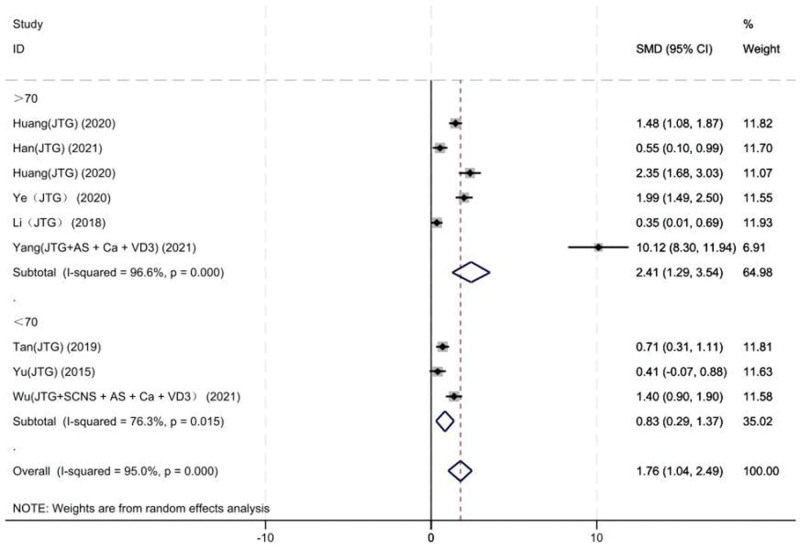
Forest plot of lumbar spine bone density subgroup analysis. AS = alendronate sodium, Ca = calcium, JTG = Jintiange capsule, SCNS = salmon calcitonin nasal spray, VD3 = vitamin D3.

Subgroup analysis of BMD of the lumbar vertebrae was performed for the mean baseline of age ≥ 70 and < 70. In 3 studies,^[15, 16, 22]^ age baseline levels were, respectively, 65.88, 67, and 68.8, which in the remaining 6 studies were all higher than 70. The heterogeneity analysis suggested that there was lower heterogeneity after subgroup analysis. The results suggested that the age may be a source of heterogeneity (Fig. 14).

The large heterogeneity of ODI scores, BMD of the femoral neck, BGP level, and ALP level may be due to the small number of trials reporting these indicators, which suggested that the results of these indicators were unstable and need to be interpreted with caution.

## 4. Discussion

Osteoporotic fracture represents a systemic bone metabolic disorder prevalent among postmenopausal women and the elderly, characterized by bone mass depletion, heightened bone fragility, and microstructural deterioration.^[26]^ Historically, long-term bed rest and pharmaceutical interventions were commonly employed in managing OVCFs, albeit with suboptimal outcomes. However, in recent years, advancements in medical technology have led to the adoption and promotion of minimally invasive treatment modalities, notably PVA, in OVCF patient care.^[27]^ Nevertheless, clinical observations reveal that while vertebroplasty yields immediate therapeutic benefits for patients with localized vertebral fractures, some individuals with primary osteoporosis continue to experience residual pain. Moreover, diminished vertebral bone mineral density and other factors predispose these patients to adjacent vertebral refractures.^[28]^ Traditional treatments often involve prolonged supplementation with calcium and vitamin D3 following therapy, yet their efficacy is frequently limited. In response, traditional herbal remedies have emerged as complementary and alternative options for osteoporosis management. The primary component of JTG capsule is artificial tiger bone powder, heralded as China’s inaugural novel drug for osteoporosis treatment.^[29]^ Renowned for its bone-strengthening and pain-alleviating properties, artificial tiger bone powder shares a similar composition with natural tiger bone.^[30]^ Numerous clinical studies have demonstrated JTG capsule’s efficacy in enhancing bone mineral density in the lumbar spine and femoral neck, thereby exhibiting promising therapeutic outcomes for osteoporosis.^[31,32]^ However, uncertainties persist regarding the efficacy and adverse effects of JTG capsules following PVA. Consequently, this meta-analysis represents the inaugural endeavor to investigate the efficacy and potential side effects of JTG capsule post-PVA.

In this study, comparing the JTG group with the Western medicine group, findings from 6 randomized controlled trials revealed a significant improvement in the total effective rate of JTG, with postoperative symptoms of OVCF patients notably alleviated within 3 to 12 months. Regarding adverse reactions, 3 studies indicated a lower incidence of adverse events (such as nausea, vomiting, dizziness, and constipation) with JTG capsule compared to alendronate combined with calcium,^[18, 19, 25]^ while one study reported a lower incidence of adverse reactions compared to calcium intake alone.^[24]^ However, when compared with the nondrug group,^[15]^ 3 cases of constipation were noted in the JTG capsule group, though it remains unclear whether this adverse reaction is directly related to JTG. In terms of the VAS score, due to significant heterogeneity in results, subgroup analysis was conducted. Subgroup analysis indicated that 5 studies^[17–19, 22, 23]^ demonstrated a significant decrease in VAS scores among OVCF patients from 3 to 12 months posttreatment, albeit with no significant difference compared to the Western medicine group within the initial 3 months posttreatment. This suggests that JTG capsule effectively alleviates postoperative pain in patients. Regarding the ODI score, 3 studies^[15, 19, 23]^ revealed that after 4 weeks of treatment, the ODI score in the JTG capsule group surpassed that of the Western medicine group, indicating effective reduction in pain, stiffness, and difficulty in daily activities among patients. However, due to the limited number of included studies, heterogeneity in some outcome measures was observed. Therefore, further large-scale clinical trials are warranted to validate these findings. Overall, the study results indicate that JTG capsule mitigates pain and enhances postoperative function, with no significant liver or kidney damage observed apart from mild gastrointestinal reactions. Additionally, the incidence of adverse reactions was lower in the JTG group compared to the Western medicine group.

Some trials included in this study also conducted comparisons of BMD and BGP among patients. The findings revealed that the BMD and BGP levels in the lumbar vertebrae and femoral neck of the JTG capsule group were higher than those in the Western medicine group. However, due to the limited number of trials, although the femoral neck BMD and BGP in the JTG group were significantly higher than those in the Western medicine group, heterogeneity was observed to be higher. Therefore, additional large-scale clinical trials are imperative to further substantiate our findings.

Clinically, JTG capsules are widely utilized for treating various common orthopedic conditions, including osteoporosis, fractures, and rheumatoid arthritis. Notably, in 2017, it was endorsed as an effective treatment in the Chinese guidelines for managing osteoporotic fractures.^[33]^ Despite this recognition, the clinical efficacy of JTG capsules in patients with OVCF following PVA lacks support from large randomized controlled trials. Both clinical and pharmacological investigations^[34,35]^ have indicated that JTG capsules can modulate the expression of osteocyte proteins and matrix metalloproteinase 3, thereby influencing bone and cartilage metabolism and effectively alleviating pain and other clinical symptoms. Modern pharmacological research suggests that artificial tiger bone, a key component of JTG capsules, is abundant in calcium, enhancing bone toughness and density. Furthermore, artificial tiger bone contains various factors crucial for bone growth, supplying ample nutrition to chondrocytes, improving their metabolism, and impeding degenerative diseases.^[9,10]^ Moreover, the active constituents of JTG capsules, namely artificial tiger bone and natural tiger bone, exhibit essentially identical pharmacological effects with heightened safety profiles, contributing to the lower adverse reaction rates observed in the experimental group.

## 5. Limitations

Certainly, it is important to acknowledge the limitations of this research. First, the quality of some included studies may be compromised due to unclear allocation concealment, lack of blinding of participants and personnel, and absence of blinding in outcome assessments. This could potentially affect the overall reliability of the findings. Second, despite efforts to conduct an unbiased literature search without language restrictions, all studies reviewed were conducted in China and published in Chinese. The absence of relevant foreign studies could introduce bias and limit the generalizability of the findings. Third, few studies provided comprehensive information on adverse reactions during or after treatment, and none reported long-term follow-up data, leaving the long-term safety of the intervention uncertain. As a result, although this study suggests that JTG capsules may have a beneficial effect on BMD and BGP concentrations in OVCF patients, the high heterogeneity among the included studies necessitated subgroup analysis. Upon exclusion of 3 studies, it was observed that JTG capsules may enhance bone mineral density in patients aged over 70 years more effectively than the Western medicine group. This implies that JTG capsules could potentially help prevent postoperative refracture in OVCF patients. However, these conclusions should be interpreted with caution due to the aforementioned limitations, and further research, particularly large-scale trials with robust methodologies and long-term follow-up, is warranted to validate these findings.

## 6. Conclusions

The findings of this meta-analysis suggest that JTG capsules hold promise in alleviating pain among OVCF patients following PVA, enhancing lumbar and back function, and improving postoperative vertebral BMD and serum BGP levels. Consequently, JTG capsules could potentially emerge as a viable treatment option for managing osteoporosis post-PVA. Nevertheless, to validate this mechanism and ensure its clinical efficacy, multicenter trials with larger sample sizes are imperative. Such studies will provide more robust evidence to support the utilization of JTG capsules in this context and further refine treatment protocols for patients undergoing PVA for OVCF.

## Author contributions

**Data curation:** Ningning Feng, Jianbin Guan, Ziye Qiu, Yongdong Yang.

**Formal analysis:** Ningning Feng, Jianbin Guan, Guozheng Jiang.

**Writing – original draft:** Ningning Feng.

**Writing – review & editing:** Ningning Feng.

**Supervision:** Xing Yu.

**Software:** Wenhao Li.
